# Intelligent indoor metasurface robotics

**DOI:** 10.1093/nsr/nwac266

**Published:** 2022-11-24

**Authors:** Hanting Zhao, Shengguo Hu, Hongrui Zhang, Zhuo Wang, Hao Dong, Philipp del Hougne, Tie Jun Cui, Lianlin Li

**Affiliations:** State Key Laboratory of Advanced Optical Communication Systems and Networks, School of Electronics, Peking University, Beijing 100871, China; State Key Laboratory of Advanced Optical Communication Systems and Networks, School of Electronics, Peking University, Beijing 100871, China; State Key Laboratory of Advanced Optical Communication Systems and Networks, School of Electronics, Peking University, Beijing 100871, China; State Key Laboratory of Advanced Optical Communication Systems and Networks, School of Electronics, Peking University, Beijing 100871, China; Center on Frontiers of Computing Studies, School of Computer Science, Peking University, Beijing 100871, China; The French National Centre for Scientific Research (CNRS), University of Rennes, Rennes F-35000, France; State Key Laboratory of Millimeter Waves, Southeast University, Nanjing 210096, China; State Key Laboratory of Advanced Optical Communication Systems and Networks, School of Electronics, Peking University, Beijing 100871, China

**Keywords:** intelligent metasurfaces, intelligent robotics, edge devices, IoT, 6G

## Abstract

Intelligent indoor robotics is expected to rapidly gain importance in crucial areas of our modern society such as at-home health care and factories. Yet, existing mobile robots are limited in their ability to perceive and respond to dynamically evolving complex indoor environments because of their inherently limited sensing and computing resources that are, moreover, traded off against their cruise time and payload. To address these formidable challenges, here we propose intelligent indoor metasurface robotics (I2MR), where all sensing and computing are relegated to a centralized robotic brain endowed with microwave perception; and I2MR’s limbs (motorized vehicles, airborne drones, etc.) merely execute the wirelessly received instructions from the brain. The key aspect of our concept is the centralized use of a computation-enabled programmable metasurface that can flexibly mold microwave propagation in the indoor wireless environment, including a sensing and localization modality based on configurational diversity and a communication modality to establish a preferential high-capacity wireless link between the I2MR’s brain and limbs. The metasurface-enhanced microwave perception is capable of realizing low-latency and high-resolution three-dimensional imaging of humans, even around corners and behind thick concrete walls, which is the basis for action decisions of the I2MR’s brain. I2MR is thus endowed with real-time and *full*-context awareness of its operating indoor environment. We implement, experimentally, a proof-of-principle demonstration at ∼2.4 GHz, in which I2MR provides health-care assistance to a human inhabitant. The presented strategy opens a new avenue for the conception of smart and wirelessly networked indoor robotics.

## Teaser

An intelligent indoor metasurface robotic is empowered on the physical layer by programmable metasurfaces and on the cyber layer by artificial-intelligence tools.

## INTRODUCTION

The wireless networked control of robotic systems is expected to fundamentally change the functioning of important areas of our civilization, including our factories, at-home health care and mobility. Many of these developments are scheduled to be rolled out within the next decade or so in the context of the 6G networks [1,2]. Wirelessly networked robotics will require an intelligently orchestrated confluence of context-awareness acquisition (localization and sensing (posture, etc.) of all human and robotic subjects) and communications (high-capacity wireless links between robotic entities and a centralized control unit) under challenging indoor conditions (strong multipath propagation for microwaves and blocked lines of sight), limited power and computation resources at the robotic edge, and an obligation to respect people's privacy. To date, context awareness in indoor robotics has been mainly acquired on the robotic edge, based on sensors in the visible or infrared part of the electromagnetic spectrum (optical cameras, lidar, etc.). This approach has numerous inconveniences. First, visual sensors have a limited field of view and can usually only operate in line of sight; they usually cannot look around corners or through typical occlusions like cardboard, wooden walls or opaque plastic layers. Recent reports show that looking around corners with optical sensor modalities [3–5] is severely limited in terms of the achievable imaging range (often below 1 m), the field of view and the dynamic range, making them unsuitable for the acquisition of context awareness in indoor robotics. Second, visual sensors cannot operate in darkness and may be sensitive to skin color. Third, by yielding human-interpretable data, visual sensors tend to infringe humans’ privacy. Fourth, operating such sensors on the robotic edge can severely limit the cruise time of battery-powered mobile robots due to power-hungry data acquisition and processing. Fifth, the acceptable payload of mobile robots, especially airborne drones, is limited. Therefore, multiple formidable challenges remain to be addressed before robots can ‘see’ and ‘understand’ a complex indoor context in order to ‘help’ humans in a future human–robot alliance.

**Figure 1.**
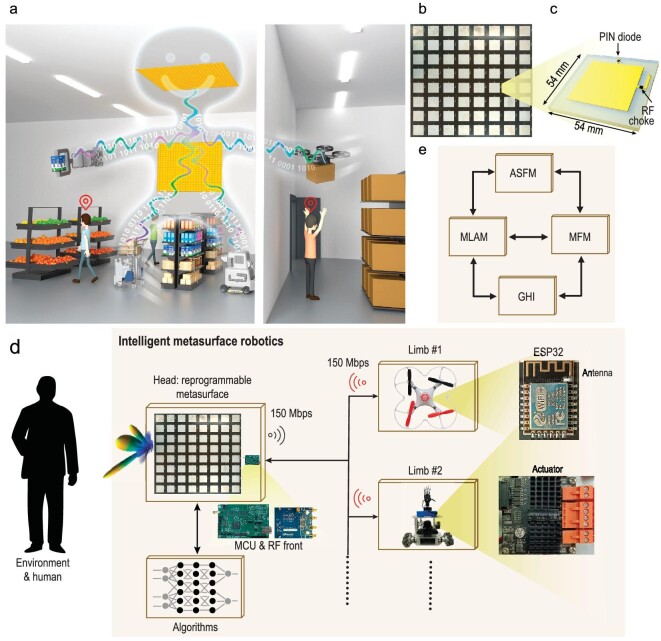
Conceptual illustration and system configuration of the proposed intelligent indoor metasurface robotics. (a) Artistic sketch of the proposed intelligent indoor metasurface robotics paradigm. Therein, robotic limbs (in the sketch, a robotic arm, two motorized robotic vehicles and an airborne drone) execute tasks as instructed by the central robotic brain. The latter combines programmable-metasurface hardware with AI software to achieve microwave perception of the complex setting (including through the wall), to plan the necessary actions of its robotic limbs and to create high-capacity wireless links to the robotic limbs to communicate the instructions. (b) Photographic image of a programmable metasurface panel with 8 × 8 meta-atoms. (c) Detailed design of a programmable meta-atom unit involving a PIN diode to enable a 1-bit programmable reflection coefficient. (d) System-level overview. The pivotal hardware ingredient is a programmable metasurface, which is controlled by a set of intelligent algorithms. A software defined radio (SDR) device (AD9361) is used to illuminate the programmable metasurface, which is intelligently deployed by the ‘robotic brain’ to sense the environment and to send wireless commands to the ‘robotic limbs’ with a communication rate of 150 Mbps. The latter are equipped with Wi-Fi modules to receive the microwave waves radiated by the SDR and modulated by the metasurface, ensuring a robust and stable wireless connection between the robotic brain and multiple robotic limbs. Besides, the Wi-Fi module on the limb will periodically emit the ACK frame radio signal to the robot's brain at a rate of 150 Mbps. The micro-controllers (ESP 32) on the limbs decode the commands received from the brain to command the actuators (electric machinery) to achieve various desired functions. (e) Signal flow in the algorithmic framework of our intelligent indoor metasurface robotics paradigm, including a general hardware interface (GHI), a metasurface functional module (MFM), a machine-learning-algorithm module (MLAM) and an application-specific module (ASFM).

To meet the requirements of intelligent wirelessly networked indoor robotics, we here demonstrate a revamped indoor robotics paradigm in which the notion of robot is distinct from the conventional definition: in our concept, robotic units like motorized vehicles, robotic arms or airborne drones are ‘limbs’ of the robot that wirelessly receive instructions from a central control unit (the ‘brain’)—see Fig. 1a. Analogous to the human body, the limbs only execute tasks following the instructions from the brain, but the limbs do not sense or process sensed data, such that they consume much less energy, which boosts their cruising time if they are battery powered. Context awareness is hence not acquired on the robotic edge, but centrally by the brain. This centralization is enabled by a change of sensing modality, instead of optical sensors with the above-listed problems, and hence we monitor the environment using microwave perception. Microwaves are predestined to meet the demands of indoor robotics in terms of operating remotely, irrespective of the illumination condition, being able to penetrate visually opaque layers like walls to see around corners, and respecting privacy by not creating images of the environment that reveal privacy-infringing visual details. To achieve high-resolution *in-situ* microwave sensing, the pivotal hardware ingredient of our concept is a programmable metasurface capable of sculpting the indoor wireless signal propagation. The programmable metasurface is an ultrathin array of meta-atoms with individually programmable scattering properties (here, their reflection coefficient) [6]; it allows the robot's brain to swiftly sense the indoor environment with configurational diversity or to reallocate microwave energy onto a desired focal spot. The latter helps to highlight features of a region of interest (ROI) while minimizing the contributions of irrelevant reflections from furniture, etc. At the same time, the ability to focus can also boost the achievable information transfer rate on wireless channels, which is important for communications between the robot's brain and limbs. Overall, the brain must make intelligent use of the available programmability of the radio environment and plan a complex sequence of tasks to instruct the robotic limbs based on the brain's understanding of the current situation.

Given its multidisciplinary nature, our approach naturally relates to various current research tracks. The potential of microwave-based perception to overcome the above-mentioned limitations of visual sensors has been an active area of research for almost a decade. On the one hand, impressive results on localization and posture recognition of humans in complex indoor settings have been achieved solely based on body reflections probed by simple microwave antenna arrays [7–10]—and hence without the subject's cooperation by carrying a tag or radio source. However, the limited degrees of freedom and small aperture of the utilized antenna arrays limited the achievable precision. On the other hand, objects were equipped with cheap radio frequency identification system (RFID), tags to enable robotic grasping of occluded objects [11–13] or to localize robots in indoor settings [14]. However, the required cooperation of carrying a tag and (again) the restricted wave control due to small antenna arrays are limiting factors. By utilizing large-aperture antenna arrays with many elements, each individually controllable in phase and amplitude, the precision of these microwave-perception schemes could be substantially boosted. But the associated hardware cost is prohibitive. Our programmable metasurfaces enable such large-aperture coherent control with a single feed at almost negligible cost.

In parallel, the field of metamaterial-enabled computational imaging has emerged since 2013 [15]. The core idea is to replace costly antenna arrays with cheap metamaterial hardware. Compressive computational imaging based on the configurational diversity of the programmable metasurfaces was introduced in refs [16,17] and was subsequently refined in terms of custom-tailored structured illuminations [18–21] (see refs [22,44] for recent comprehensive reviews). However, these investigations were limited to stand-alone imaging applications in free space rather than being integrated into a robotic control algorithm operating in a complex multipath indoor environment. Moreover, the feasibility of using programmable metasurfaces for object localization has been investigated separately from imaging and sensing based on programmable metasurfaces both in free space [23,24] and rich-scattering environments [25]. Meanwhile, limited resources encourage increasing interest in integrated sensing and communication (ISAC) functions in the wireless communication community [26–28]. Recently, the importance of sensing to enable wireless communication in dynamic smart metasurface-programmable radio environments has received much attention [29–34]: in order to know how to configure the metasurface to assist the communications, one must identify the current configuration of the wireless environment. However, except for ref. [34], these investigations are still limited to echo-free anechoic environments, which do not capture the complexity of targeted realistic rich-scattering indoor environments like warehouses, factories, hospitals and homes [34]. Especially in the microwave regime, indoor environments give rise to rich scattering, as seen in the experimental studies [35–37]. Note that we deliberately chose the microwave regime because this scattering allows us to sense around corners, and the reverberation also improves the sensing and localization resolution [38]. However, the significant multipath propagation also implies that non-intuitive metasurface coding patterns are required for focusing that cannot be derived analytically based on a free-space assumption.

In this paper, we prototype an intelligent cyber-physical robotic system in the context of ambient assisted living for smart health care at home, empowered on the physical layer by programmable metasurfaces and on the cyber layer by artificial-intelligence tools. Our system operates at ∼2.4 GHz. The robot's brain performs a complex sequence of sensing tasks to locate its mobile robotic limb as well as a human, and to recognize the human's posture. Based on the human posture, the brain identifies the necessary action of the limb. The brain then implements a high-capacity communication link with the robotic limb and transmits the instructions. We envision a smart human–robot alliance built upon our intelligent indoor metasurface robotics paradigm. Our concept may also be adapted and transposed to other length scales and wave phenomena, e.g. light-driven microscopic meta-vehicles [39,40].

## SYSTEM CONFIGURATION

A system-level overview of our intelligent indoor metasurface robot (I2MR) is shown in Fig. 1d, and comprises two main entities. The first entity is the ‘brain’ equipped with a metasurface-centered microwave perception modality and an AI-empowered data processor. The I2MR’s brain is powered by a socket. The second entity of I2MR are its ‘limbs’, such as motorized ground-based vehicles or airborne drones, which are powered by on-board batteries. The I2MR interacts with its environment and the humans therein by, first, using its brain to acquire full context awareness and to decide what action is needed; and, second, using its limbs to mechanically perform these actions in the environment. The limbs are thus not performing any sensing or ‘thinking’ (interpretation of sensing data to determine necessary actions) tasks, meaning that the on-board battery lasts longer, and the available payload is larger. Instead, the I2MR’s AI brain processes the sensor data that the brain acquires in real time to instruct the limbs how to act. If the brain concludes that action is required, it localizes its limbs and establishes a wireless link to transmit instructions at a rate of 10 Hz. The limb will then perform the demanded action via an ESP32 controller that is embedded with a 2.4 GHz Wi-Fi module based on IEEE 802.11 b/g/n standards.

The brain's microwave perception, as well as the wireless communications between brain and limbs, pivotally rely on a programmable metasurface that is part of the brain. The metasurface's large aperture and massive number of degrees of freedom are used by the I2MR’s brain sequentially in two different manners: first, to generate configurational diversity in order to illuminate the indoor environment with pseudo-random patterns for sensing and localization, and, second, to focus microwaves on the limb's wireless receiver to create a preferential high-capacity wireless link with boosted received-signal-strength indicator (RSSI). In our experiment, the programmable metasurface is spatially distributed in two locations: $4 \times 3$ metasurface panels are installed parallel to a wall, and an additional $3 \times 3$ metasurface panels are installed on the ceiling (see Supplementary Fig. 1). A metasurface panel is composed of $8 \times 8$ meta-atoms each with a 1-bit programmable reflection coefficient around the frequency of 2.4 GHz. A photographic image of an $8 \times 8$ panel of these meta-atoms is shown in Fig. 1b. As seen in Fig. 1c, the meta-atoms consist of a sandwiched structure with a PIN diode embedded on the top layer. The bias voltage of this PIN diode determines the reflected phase upon illumination with a linearly polarized plane wave in normal incidence: in state ‘1’ there is a 180° phase shift whereas for state ‘0’ there is a 0° phase shift. Further details about the metasurface design are provided in Methods and Supplementary Note 1. Judiciously chosen configurations of the programmable metasurface can flexibly manipulate the electromagnetic waves in the entire indoor environment. The I2MR’s brain controls the metasurface configuration via an field programmable gate array (FPGA)-based micro-control unit (MCU) with a clock of 766 MHz. Microwave signals are generated and acquired using a radio frequency (RF) transceiver module (AD9361); the latter is connected to two pairs of commercial planar antennas, as well as to the FPGA through an FPGA Mezzanine Card for synchronization purposes. On the software level, I2MR operates in a Python environment through a complex sequence of AI-empowered algorithms, as detailed in Supplementary Note 2. In the following sections, we discuss each of these algorithmic steps and their interplay in detail.

## CENTRALIZED ACQUISITION OF CONTEXT AWARENESS

The first step for our I2MR is to acquire full context awareness in order to be able to subsequently make informed decisions about necessary actions. As stated previously, our I2MR paradigm is based on a centralized microwave-perception sensing modality that is part of the robot's brain—as opposed to the conventional approach of integrating optical sensing modalities into the robot's limbs. The centralization of I2MR’s microwave perception in the robot's brain is enabled by the ability of microwaves to penetrate through walls and other visual obstacles, in combination with the configurational diversity offered by the programmable metasurface to shape the microwave fields into a series of pseudo-random patterns that illuminate the indoor environment. The ‘context’ of which our I2MR should be aware is the posture and location of the human inhabitant of the indoor environment under consideration. Specifically, the human's posture may reveal information about health status (e.g. illness, or a fall or accident) or explicit action commands from the human that are conveyed through body gestures.

**Figure 2.**
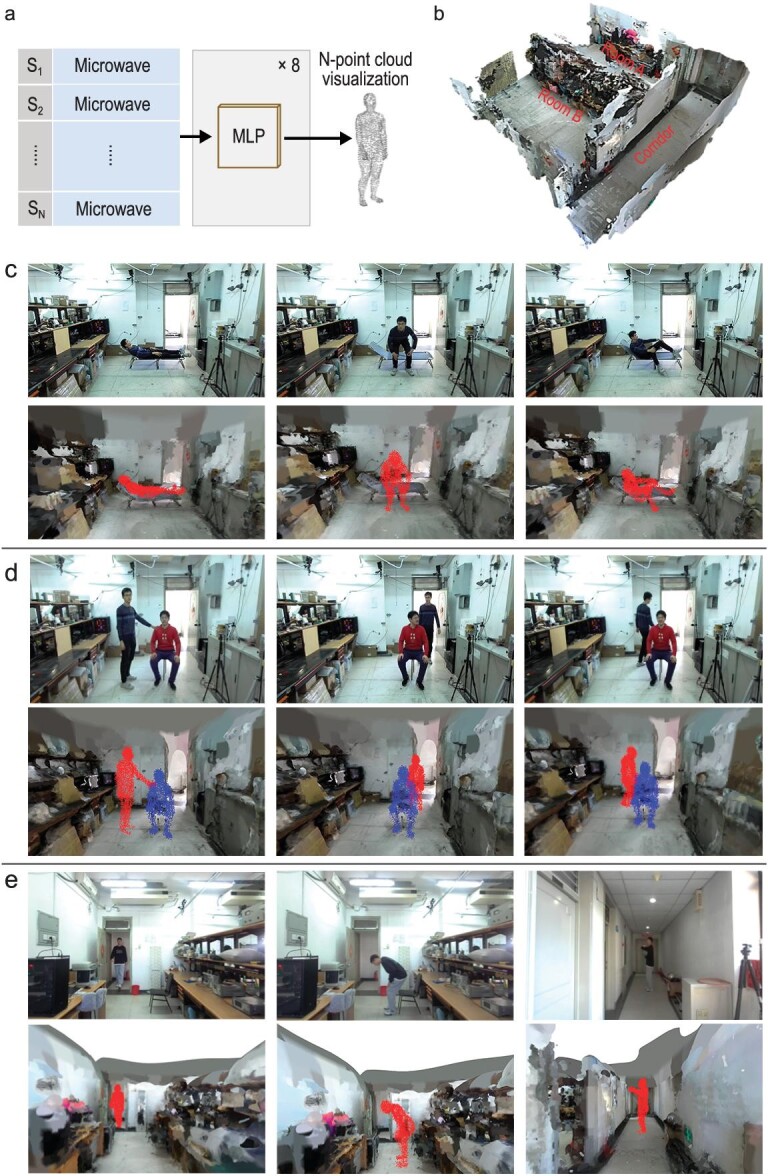
Experimental results of *in-situ* human sensing with the developed metasurface robotics. (a) High-level overview of the point-cloud network used for the recognition function, mapping the microwave data into the N-point representation of the human body. MLP: multiple layer preceptor; S_i_: *i*th point of the prior point-cloud representation; microwave: the 64-length reduced microwave measurements from the raw data with a size of 20 × 128, where 20 is the number of metasurface coding patterns and 128 is the number of frequency points with which the observed signal is sampled for each metasurface pattern. Notice that Microwave is duplicated N times for the N cloud points. Additional details are provided in Supplementary Note 3. (b) Point-cloud representation (obtained using an optical ZED camera) of the experimental indoor environment with Room A, Room B and a corridor. There is a 60-cm-thick concrete wall between the rooms and the corridor. Room A is equipped with two programmable metasurfaces: 24 × 32 meta-atoms are installed on a wall, and another 24 × 24 meta-atoms are installed on the ceiling (hence not seen). (c) Representative line-of-sight *in-situ* sensing results for a single person in Room A. The photographic image is printed as ground truth in the first line, and the reconstructed 3D point-cloud representations are shown in the second line. (d) Representative line-of-sight *in-situ* sensing results for two humans simultaneously present in Room A. (e) Representative non-line-of-sight *in-situ* sensing results when one human is in Room B or in the corridor.

The basis of I2MR’s microwave perception is to illuminate the indoor environment with a fixed series of 20 random coding patterns of the programmable metasurface, and to reconstruct a 3D point-cloud model of the human body. This use of the configurational diversity of the programmable metasurface differs in two important aspects from refs [16,17], which first used configurational diversity for computational meta-imaging. First, I2MR uses an algorithm that directly maps the measured microwave data to a 3D point cloud, without an intermediate image-reconstruction step. Further details about the network architecture are provided in Fig. 2a and Supplementary Note 3. Second, I2MR operates in a highly challenging indoor environment whereas refs [16,17] imaged a scene in free space. In free space, the Green's functions between transceiver coordinates and scene coordinates are analytically known. In contrast, given the complex geometry of our indoor environment, an analytical description of the Green's function is intractable. Instead, our AI-based reconstruction algorithm implicitly learns the Green's functions from the training data, in some ways similar to refs [21,41]. Specifically, to obtain a large labeled data set to train our network with supervised learning, we use a set of stereo optical cameras that are embedded in the environment and synchronized with the I2MR during training. To be clear, these cameras are only used during training. During training, 10 participants (3 females and 7 males) were asked to act freely in our indoor environment seen in Fig. 2b. In total, we have collected $8 \times {10}^8$ samples of labeled training data, and it took around one week to train the recognition network. During runtime, the trained network is now capable of producing high-resolution images of a test person in less than 1 microsecond. All computations are performed on a small-scale server with 128 GB memory, an Intel Core i7-7820X central processing unit, and an NVIDIA RTX 2060 GPU.

Representative results of our high-resolution indoor human sensing are displayed in Fig. 2c–e. In particular, we demonstrate that I2MR’s microwave perception of a human context also works successfully when there are multiple interacting humans in the indoor environment (Fig. 2d), who can partially shadow each other, as well as under non-line-of-sight conditions when the human is behind a 60-cm-thick concrete wall (Fig. 2e). Throughout these experiments on capturing human postures, the mobile vehicle is present. Its motion during runtime constitutes essentially a minor perturbation to which the imaging algorithm is robust [42]. Based on the high-resolution 3D point clouds, I2MR’s brain can interpret the human's posture, analyzing its well-being and identifying potential explicit commands through body language. Before closing this section, we note that, in principle, judiciously structured metasurface configurations may be capable of reducing the number of required waveforms in order to answer a specific task, and hence improve latency [20–22]. This is left as an idea for future work on I2MRs.

## INTEGRATED LOCALIZATION AND COMMUNICATIONS

Once our I2MR has gained context awareness and decided to take action, it must locate and instruct its limb(s). In our experiments, the limb is a motorized commodity vehicle whose computing and sensing capabilities are shut off. Since our I2MR paradigm does not require sensing or computing resources in the robot's limbs, it is generally very easy to integrate many other resource-limited edge devices into the robot's collection of limbs. The robot's brain intelligently uses the programmable metasurface hardware to firstly locate the limb and then establish a high-capacity wireless link for wireless communication. This time-sequential sharing of the same hardware for localization and sensing is a typical instance of ISAC. For simplicity, we consider a conventional wireless communication scheme, in which the transmitter uses a software defined radio (SDR AD9361) to generate a radio signal and encode information into it. The role of the programmable metasurface is to shape the wireless channel to create a constructive interference at the receiver that boosts the RSSI and hence the achievable communication rate. Future work can use the programmable metasurface to directly encode the digital information into stray ambient waves in a massive backscatter wireless communication scheme to improve various metrics including energy consumption and spectrum allotment [43] (see also ref. [44] for a recent review).

**Figure 3.**
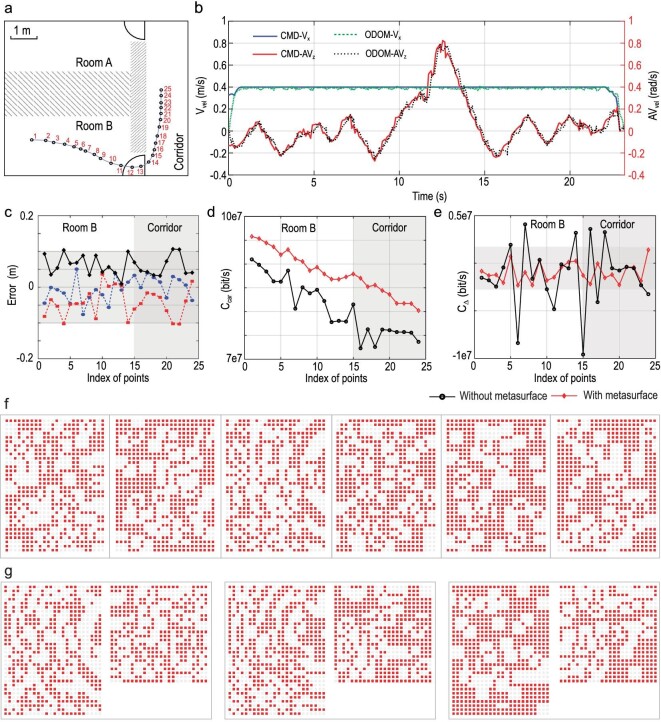
Experimental results of the localization and communication of I2MR. (a) Sketch of the indoor environment's layout, highlighting the trajectory over which the motorized vehicle (size: 40 cm × 40 cm × 25 cm) navigates through a narrow door (width: 87 cm). (b) Linear and angular velocity measured by the in-built odometry of the mobile vehicle (ODOM-V_x_ and ODOM-AV_z_), and the linear and angular velocity instructions sent from the metasurface brain (CMD-V_x_ and CMD-AV_z_). (c) Localization error in terms of x-coordinate (blue line), y-coordinate (red line) and the Euclidean distance (black line) for the trajectory seen in (a). The ground truth of the position of the limbs is obtained with an eight-camera NOKOV motion capture system to which an associated 7.5-mm-radius optically sensitive ball is attached. (d) Communication capacity ${C}_{{\rm{car}}}( n )$ of the wireless link between the limb and the brain for the trajectory seen in (a). (e) Differential information capacity ${C}_\Delta \ ( n ) = {C}_{{\rm{car}}}\ ( n ) - {C}_{{\rm{car}}}( {n - 1} )$ for the trajectory marked in (a). (f) Six control coding patterns used to localize the robot's limb for the programmable metasurface on the wall. A red-filled (white) square denotes that a meta-atom is in its ON (OFF) state. More results are provided in Supplementary Note 4. (g) Three optimized pairs of control coding patterns of the programmable metasurfaces on the wall (left pattern) and ceiling (right pattern) for the boosted wireless link connecting the robot's brain with the limb. The four patterns correspond to the three points indexed 10, 11 and 12 of the limb's trajectory marked in (a). More results are provided in Supplementary Note 5.

Given the complex multipath propagation in the indoor environment, the localization of the robot's limb by leveraging the programmable metasurface in combination with localization algorithms based on a free-space assumption and ray tracing (see e.g. refs [23,24]) is unfeasible. However, the complex scattering can be understood as a ‘reverberation-coded aperture’ [38] that multiplexes the sought-after location information across a series of measurement modes (pseudo-random metasurface configurations) onto a single receiver. The captured corresponding scattered signals are hence like a wave fingerprint of the object location [25], and strong reverberation can considerably boost the achievable localization precision because it provides interferometric sensitivity [38]. Following these insights from refs [25,38], the I2MR’s brain receives the acknowledge character (ACK) frame signal emitted from the Wi-Fi module of the robot's limb (see below) using a fixed series of 20 random metasurface configurations, and localizes the robot's limb based on the corresponding measurements. Six of the utilized configurations of the metasurface on the wall are shown in Fig. 3f. The measured data are processed by a simple complex-valued long short-term memory recurrent neural network (LSTM RNN) to unscramble the wave fingerprint and infer the limb's location (see Supplementary Note 4 for additional details). This RNN is trained using a training data set of 20 000 samples that are collected for random locations of the robot's limb in the entire indoor environment (Room A + Room B + corridor). These training samples are collected while the human is simultaneously present at random locations such that the RNN can learn to robustly localize the limb despite the perturbing motion of the human [42]. In Fig. 3a–c, we navigate the robot's limb through a narrow door while the robot's brain constantly localizes its limb. The results reveal that we achieve a very high localization precision on the order of a few millimeters. This localization precision is hence well below the wavelength even though we do *not* capture any evanescent waves in the limb's close vicinity. This extraordinary precision can be attributed to both our extensive a priori knowledge, included in the training data, as well as to the fact that multipath propagation boosts the achievable precision through interferometric sensitivity (see ref. [38]).

Once the limb is localized, the brain must efficiently transmit instructions for the required actions. Therefore, a high-capacity wireless channel must be established between brain and limb. We achieve this through a metasurface configuration that focuses microwaves on the limb's receiving antenna in order to boost the RSSI [36] and thereby the SNR and ultimately the channel capacity. Due to the strong multipath nature of the indoor environment, free-space equations cannot be used to determine the best metasurface configuration for this focusing purpose. Instead, an offline calibration to the specific indoor environment is necessary. As discussed in ref. [34], this can be an open-loop calibration (learning a surrogate forward model to predict how the metasurface parametrizes the wireless channel) or a closed-loop calibration (establishing a code book that pre-stores the optimal pattern for each possible location of the robot's limb on a two-dimensional grid). The former can be very challenging to implement and use during runtime, so we opt for the closed-loop approach here with a square grid spacing of 0.1 m. The identification of the optimal configuration for a given location of the robot's limb requires an iterative trial-and-error algorithm [34,36] (see Supplementary Note 5 for details). In Supplementary Note 5, 12 optimized pairs of control coding patterns of the programmable metasurfaces on the wall and ceiling are reported, corresponding to the first 12 points of the planned limb's trajectory seen in Fig. 3a. Three pairs of coding patterns corresponding to the points indexed 10, 11 and 12 of the planned trajectory are plotted in Fig. 3g. In Fig. 3d, we quantify the channel capacity improvement thanks to the optimized metasurface configuration that we choose from our code book based on our location estimate. The channel capacity at the *n*th point (*n* = 1, 2, …, 25), as marked in Fig. 3a, is defined as ${C}_{car}( n )\ = \ B{\rm{lo}}{{\rm{g}}}_2( {1 + S( n )/N( n )} )$, where *B* is the frequency bandwidth, *S* is the average power of the received signal and *N* is the measured noise power. The specific values of *S* and *N* are implicitly dependent on the current limb location (parametrized by the point index *n*). On average, we improve the channel capacity by roughly $5 \times {10}^6$ bits per second, despite the challenging indoor conditions; recall that the trajectory in Fig. 3 includes non-line-of-sight scenarios where a visually opaque 60-cm-thick concrete wall separates the robot's brain and limb. The wireless channel between the brain and the moving limb is a short-term fading wireless channel. To quantify the variation of the wireless communication quality, we define the differential capacity ${C}_\Delta ( n )\ = {C}_{car}\ ( n ) - {C}_{car}( {n - 1} )$. We demonstrate in Fig. 3e that we manage to reduce the fluctuation of the wireless channel capacity by a factor of $9 \times {10}^6$ bits per second. Further improvements of the wireless link could be possible by using a code book with more entries for a grid with better resolution [34].

Wireless communication makes, of course, use of a finite bandwidth. In this respect, it should be noted that the focusing effect that we achieve at a single targeted frequency extends over an interval of a few tens of MHz; the specific extent depends on the composite quality factor of the room—the higher the attenuation in the room, the larger the spectral correlation length. For example, ref. [36] observed that the focusing effect was significant across a 40 MHz interval centered on the targeted frequency, which is sufficient for a Wi-Fi link. Finally, we point out that due to the rich multipath propagation, the focusing that we achieve cannot be described as ‘beamforming’, which is a free-space notion. Instead, the field will have an approximately isotropic maximum because rays from all possible angles are incident [36]. Overall, our results allow us to conclude that the combination of the programmable metasurface with ISAC algorithms allows the robot's brain to accurately localize its limbs and achieve low-latency information transfer.

## ROBOTICS–HUMAN INTERPLAY

**Figure 4.**
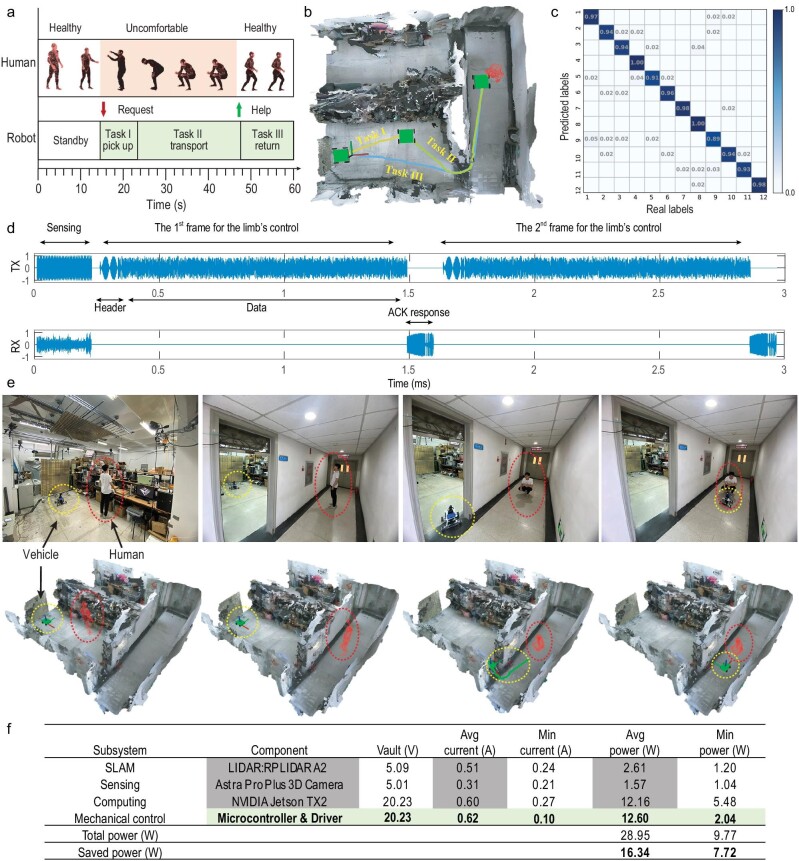
Experimental results for robot–human interaction. (a) Schematic diagram for the robot–human interaction. When the subject in the corridor exhibits an unhealthy state, the intelligent robot will automatically recognize the human's state, and perform three tasks. Task 1: the robot controls its limb to move to the medicine cabinet and reaches out its arm to grab the target drug. Task 2: the robot controls its limb to deliver the medicine to the unwell human in the corridor. Task 3: the robot controls its limb to return to the original position. (b) The trajectories of the robot's limb planned by the robot's brain, which correspond to the three tasks in (a). (c) The classification confusion matrix of 12 different body gestures, when the subject is in Room A. See Supplementary Note 6 for details. (d) 3-ms-long sample of the microwave signals emitted (top) and received (bottom) by the robot's brain. The emitted signal has two parts: one is the probing signal for sensing the subject, and the other is for transferring the command to the robot's limb via wireless communication. Here, we illustrate two command frames for the limb's control, and each frame includes two parts, i.e. header and data. The signal received by the robot's brain has two parts: one is the probing microwave signal scattered by the subject and surrounding environment, and the other is the ACK frame emitted by the Wi-Fi module installed on the robot's limb. (e) Four selected snapshots of human–robot interaction results. More complete results have been recorded in Supplementary Video 1. The top row displays ground truth optical results, while the bottom row displays the 3D point-cloud result retrieved from the microwave signal. The subject and the robot's limb (a driverless mobile vehicle) are marked with the dashed red line and yellow line, respectively. In addition, the planned trajectory for the robot's limb is represented by a green line in the third frame. (f) Analysis of the power consumption of the robot's limb. For comparison, we consider a typical commodity mobile vehicle configured with two sensors (RPLIDAR A2, Astra pro Plus 3D) and an NVIDIA Jetson TX2 processor for the SLAM (simultaneous localization and mapping), object detection and computing, respectively.

Having established how our I2MR acquires context awareness as well as how it localizes and communicates with its limb, we now examine the performance of the interplay of all these complex tasks. Our test scenario mimics a typical future robot-assisted health-care-at-home scenario, suitable, for example, for allowing elderly people to live an autonomous life in their own homes. Our I2MR monitors, via its microwave perception, the motion of a human inhabitant. At some point in time, the human pretends to feel unwell by signaling to the robot the need for a pill using the body gesture ‘start’ (see Supplementary Note 6 for different body signs) and subsequently assumes a posture that reveals unwell-being—see Fig. 4a. Once the robot has recognized the human's request for assistance, the robot determines the human's location from the 3D point-cloud representation of the human and subsequently plans the trajectory of its motorized-vehicle limb in terms of the associated parameters (speed and angular velocity). Then, I2MR localizes its limb, establishes a high-capacity wireless communication link, and wirelessly transmits the commands to be followed by the limb. The robot's limb then begins to smoothly move along the instructed trajectory under the continuous control and supervision of the robot's brain.

In order to assist the human, the robot's limb must execute the three tasks seen in Fig. 4b: first, it must rush to the medicine cabinet and grab the requested pill; second, it must deliver the medicine to the unwell human in the corridor; third, it must return to its original location. Notice that the chosen setting is particularly challenging because the human pretends to feel unwell while being outside the line-of-sight of the I2MR’s brain. The microwave signals used by the I2MR over the course of this human–machine interaction and robotic health-care assistance are shown in Fig. 4d. In particular, I2MR emits 20 continuous wave (CW) signals that are used in combination with a fixed series of 20 random metasurface configurations to acquire context awareness (location and posture of the human, see Fig. 4c and e). Body gesture recognition can be treated as a classification problem, and we solve it using an LSTM RNN, as detailed in Supplementary Note 6. To this end, we conceive 12 different body postures as shown in Supplementary Note 6 and collect 8000 samples for each body posture. Supplementary Fig. 6a and b report the recognition performance of the microwave intelligent robotics in terms of the confusion matrix when the subject is in Room A or the corridor, respectively. These results show that our intelligent robotics can recognize the body language with an accuracy of >95%, even when the subject is behind a 60-cm-thick concrete wall.

When the robot's brain recognizes the request from the subject by their body language (see details in Supplementary Note 6), it deduces the subject's location from its current 3D point-cloud representation of the environment, and it plans the motion trajectory for the robot's limb with the associated parameters (e.g. speed and angular velocity). The robot's brain sends a series of data packages to the robot's limb via the established wireless link, such that the limb can accordingly act. Meanwhile, the Wi-Fi module (802.11b/g/n protocol) integrated into the robot's limb emits the ACK frame carrying its unique media access control (MAC) address to the robot's brain. Afterwards, the robot establishes the point-to-point wireless link between its brain and limb, and then transfers, continuously, the executive commands to the limb via the established wireless link. Thereby, the robot's limb moves smoothly along the specified trajectory under the continuous control of the robot's brain, and performs the tasks assigned by the robot's brain. This experiment is documented in Supplementary Video 1, and selected snapshots are reported in Fig. 4e, where the actual scene is shown in the top row, and the corresponding contextual understanding of the robot's brain is reported in the bottom row for comparison.

Finally, we carry out an experimental analysis of the power consumption of the robot's limb in Fig. 4f. We consider a typical commodity mobile vehicle equipped with two high-end sensors and a suitable processor (RPLIDAR A2, Astra pro Plus 3D and NVIDIA Jetson TX2) to perform SLAM (simultaneous localization and mapping), object detection and computing. Apparently, more than half the power consumption is conventionally dedicated to sensing and computing. Our I2MR approach can hence save 56.4% power on the mobile robotic edge and hence more than double the battery lifetime, by relegating sensing and computation tasks to the centralized robotic brain equipped with rich sensing resources (empowered by a programmable metasurface) and computing resources. Our robot's limb only needs a microcontroller and motor driver. Thus, our I2MR supports the flexible control of a computation-free robotic vehicle in a real-time and smart way, showing promising potential for *in-situ* remote management of human–robot interplay in real-world indoor settings. The I2MR can assist humans in an unobtrusive way, and wirelessly connect people with surrounding devices in a smart way. In this sense, our strategy could yield a new intelligent interface between humans and devices, which enables devices to remotely sense and recognize complicated human behaviors at a low cost. Before closing this section, we would like to discuss briefly the safety of our centralized intelligent metasurface robot in comparison to conventional decentralized robots. The decentralized robot architecture uses the perception-action-communication architecture, where each operation (perception, action, communication) involves interaction with the surrounding environment, and suffers easily from physical-level attack. In contrast, our centralized intelligent metasurface robot can incorporate physical-level metasurface encryption strategies for perception and communication [44,45] due to the unique capability of its reprogrammable metasurface to manipulate the EM wavefield and information. In addition, our centralized robot architecture performs the operations of sensing and data processing on the level of the metasurface brain, and the communication between the metasurface brain and limbs involves only low-capacity control commands. In this sense, the data throughput involved in our robot architecture is remarkably reduced when compared with the decentralized architecture, and this can enhance the robot's safety. Therefore, safety can be fully guaranteed in our intelligent metasurface robot.

## CONCLUSION

To summarize, we introduced the concept of the I2MR, whose unconventional operating principle involves (i) a centralized robotic brain endowed with metasurface-empowered microwave perception and processing resources and (ii) remote robotic limbs that merely execute the instructions that they receive wirelessly from the brain. In this way, the cruise time and payload of motorized vehicles and airborne drones acting as robotic limbs is drastically improved because no resource-hungry sensing or computing equipment is carried. At the same time, centralized *full* context acquisition is enabled in a privacy-preserving manner through microwave (rather than optical) perception around 2.4 GHz, the performance of which is boosted through the agile multipurpose programmable metasurface hardware. The robotic brain can ‘understand’ the *full* context of an indoor environment through sensing and subsequent interpretation—even through visually opaque walls and around corners, and irrespective of lighting conditions. We have reported proof-of-principle experimental results of our I2MR paradigm in a prototypical context of robotics-assisted health care, e.g. for ambient assisted living. Our work has illustrated how the parametrization of a wireless environment through a programmable metasurface can be intelligently leveraged in robotics to perform a series of sensing, localization and communication tasks, all orchestrated by AI-empowered algorithms. Here, we would like to say that the presented methods could be extended to other frequencies and beyond for developing more intelligent robotics with more advanced functionalities. In the future, I2MR can be transposed to further important application areas of wirelessly networked robotic entities, e.g. in factories.

## METHODS

### Design of programmable metasurface

Our programmable metasurface operates around 2.4 GHz and is composed of a two-dimensional array of electronically controllable meta-atoms. Each meta-atom is equipped with a PIN diode enabling two binary states: ‘0’ and ‘1’, corresponding to two microwave response states with a 180º phase difference. More specifically, the phase response of the meta-atom changes by 180° around 2.4 GHz when the PIN diode is switched from OFF (ON) to ON (OFF). We consider two one-bit programmable coding metasurfaces: one with 32 × 28 meta-atoms is installed on the wall in Room A, and the other with 28 × 28 meta-atoms is installed on the ceiling in Room A, as detailed above. Each programmable metasurface is electronically controlled with an FPGA-based MCU. The FPGA chip is used to distribute all commands to all PIN diodes to achieve the real-time and flexible controls of the PIN diodes soldered onto the programmable metasurface. The power required to program the metasurface is minimal and can be as low as a few $\mu $W per meta-atom [36].

### Configuration of the robot's limb

In our experiment, the robot's limb is designed with the following three functional modules (see more details in Supplementary Note 7).

#### Communication module

This module enables communication between the robot's brain and limb. On the one hand, it receives instructions for the actuators from the brain. On the other hand, after each received frame of instructions, it emits an ACK frame carrying a unique MAC address. This emitted signal allows the brain to localize the limb.

#### Control module

This module is used to provide real-time processing of the control commands received from the brain, requiring only simple calculation and processing capabilities. It converts the control commands from the brain into control quantities for each actuator's drive module. Examples of control modules that satisfy the design requirements include Raspberry Pi, TX2, ESP32 and other compact micro-controllers.

#### Actuator module

This module is used to drive the actuators to perform actions and can hence be flexibly designed for the specific tasks to be performed by the robot. In general, it may include motors, servos, displays, audio equipment, etc.

### Algorithmic overview

I2MR orchestrates a multitude of AI-empowered algorithms to perceive and act in its environment. First, I2MR obtains a 3D point-cloud representation of its environment using the configurational diversity of 20 random metasurface patterns. Second, I2MR analyzes the 3D point-cloud neural network and the LSTM RNN to achieve the image and recognize the body gesture of the human subject. Third, if the need to bring medicine to the human is deduced, I2MR locates its limb using eight random metasurface configurations. Fourth, I2MR establishes a high-capacity wireless link between its brain and limb to wirelessly transfer instructions for the limb using an optimized metasurface coding pattern from a previously established code book. The location of the limb is constantly tracked, and the metasurface pattern, which is used to create a boosted wireless link, is constantly updated accordingly. Details on the limb's internal working principle are provided in Supplementary Note 7.

## Supplementary Material

nwac266_Supplemental_Files
