# Supplementary material for: Intelligent indoor metasurface robotics
Source: Natl Sci Rev. 2022 Nov 24;10(8):nwac266. doi: 10.1093/nsr/nwac266 (PMC10309179; doi:10.1093/nsr/nwac266)
Supplement: nwac266_Supplemental_Files [file nwac266_supplemental_files.zip › Supplementary data.docx]

**Supplementary Materials for**

**Intelligent Indoor Metasurface Robotics**

Hanting Zhao^1+^, Shengguo Hu^1+^, Hongrui Zhang^1+^, Zhuo Wang^1^, Hao Dong^2^, Philipp del Hougne^3^, and Tie Jun Cui^4^ , Lianlin Li^1^

^1^ State Key Laboratory of Advanced Optical Communication Systems and Networks,

School of Electronics, Peking University, Beijing 100871, China

^2^ Center on Frontiers of Computing Studies, School of Computer Science, Peking University, Beijing 1000871, China

^3^ Univ Rennes, CNRS, IETR - UMR 6164, F-35000 Rennes, France

^4^ State Key Laboratory of Millimeter Waves, Southeast University, Nanjing 210096, China

^+^ These authors contributed equally to this work.

**Supplementary Video 1:** Video of robotics-human interplay in indoor environment.

**Supplementary Note 1. I2MR’s configuration and the programmable metasurface**

Our I2MR demo system is designed, for illustration, at around 2.4GHz, aiming at a robot-human interplay in indoor environments. With support of **Suppl. Fig. 1a** and **d**, the robot’s head consists of two programmable metasurfaces (one is installed parallel to a wall, and the other is on ceiling), a host computer with GPU (3080Ti), two FPGA core boards on the back side of the metasurface, two metasurface drive circuit boards on the back side of the metasurface, a two-antenna transmitter (Tx-1, Tx-2), and a three-antenna receiver (Rx-1, Rx-2, Rx-3; note Rx-3 is on the left wall in room A and not shown here).

- Programmable metasurface

The programmable metasurface consists of an array of one-bit electronically controllable elements, called one-bit programmable meta-atoms. As shown in **Suppl. Fig. 1b**, the meta-atom has two substrate layers: the top substrate is F4B with a relative permittivity of 2.55 and a loss tangent of 0.0019, and the bottom substrate is FR4. A SMP1345-079LF PIN diode is integrated into the top square patch and connected to the ground plane via a hole. An RF choke with inductance L = 33nH is used to suppress the AC coupling to ground. We examined the EM performance of the designed meta-atom numerically and experimentally. The results from our simulations and experiments are plotted in **Suppl. Fig.1c**. It can be observed that the reflection phase of the meta-atom experiences a 180^o^ phase difference when the PIN diode is switched from ON (OFF) to OFF (ON) in the frequency range 2.41-2.48 GHz. The phase change is accomplished by switching the external DC voltage applied to the PIN diode from 3.3V to 0V.

- The FPGA core board

The FPGA (Xilinx Zynq-7020) works as the central mico-control unit (MCU) of the metasurface, which consists of an ARM Dual Cortex A9, and programmable logic resources (such as, 802.11n communication modules, peripheral synchronous clock controller (PSCC), sensing controller, metasurface controller), as shown in **Suppl. Fig. 1d**. The FPGA synchronizes all hardware modules involved in the I2MR through the PSCC. An 8GB DRAM (DDR3) is introduced and connected with the FPGA to cache the control coding sequences from the host computer. Thereby, the programmable metasurface can be switched under control of the FPGA with millisecond-level timing. Besides, the FPGA is responsible for the following roles:

1. Converting the coding sequence from the host computer into a set of serial SPI data.
2. Assigning the bit streams to the metasurface panel through the IO interface.
3. Reading the device number information from the memory chip on the metasurface ID circuit (MIDC).
4. Detecting and diagnosing any abnormal status of the metasurface.

- Metasurface drive circuit board (MDCB)

The MDCB consists of the metasurface ID circuit (MIDC), the bitstream parallel distribution circuit (BPDC) and the PIN drive circuit (PINDC). The BPDC is composed of an 8-bit serial-to-parallel shift register (74HC595), which is utilized to drive the PINDC. The PINDC is an array of double-triode push-pull circuits, which can be refreshed at a speed of 1 MHz. Each push-pull circuit uses + 5 V and -12 V voltages to provide the PIN diode forward bias and reverse bias, respectively, and to switch between these two states. Thereby, the states of the meta-atom can be switched ON or OFF in real time. The MIDC corresponds to a Flash E^2^PROM memory chip communicating with the SPI protocol, which is used to store the control coding sequence of the metasurface.


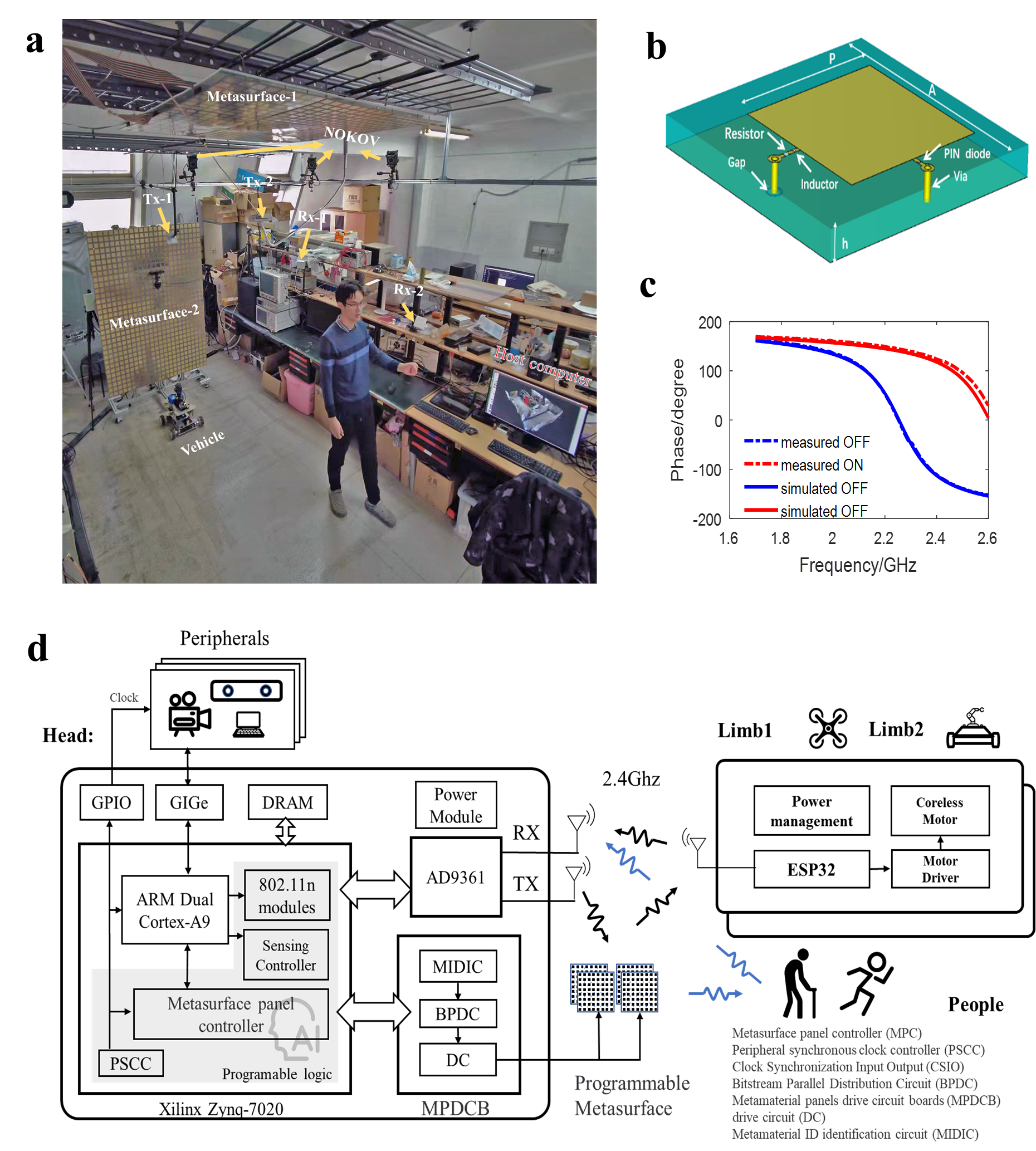

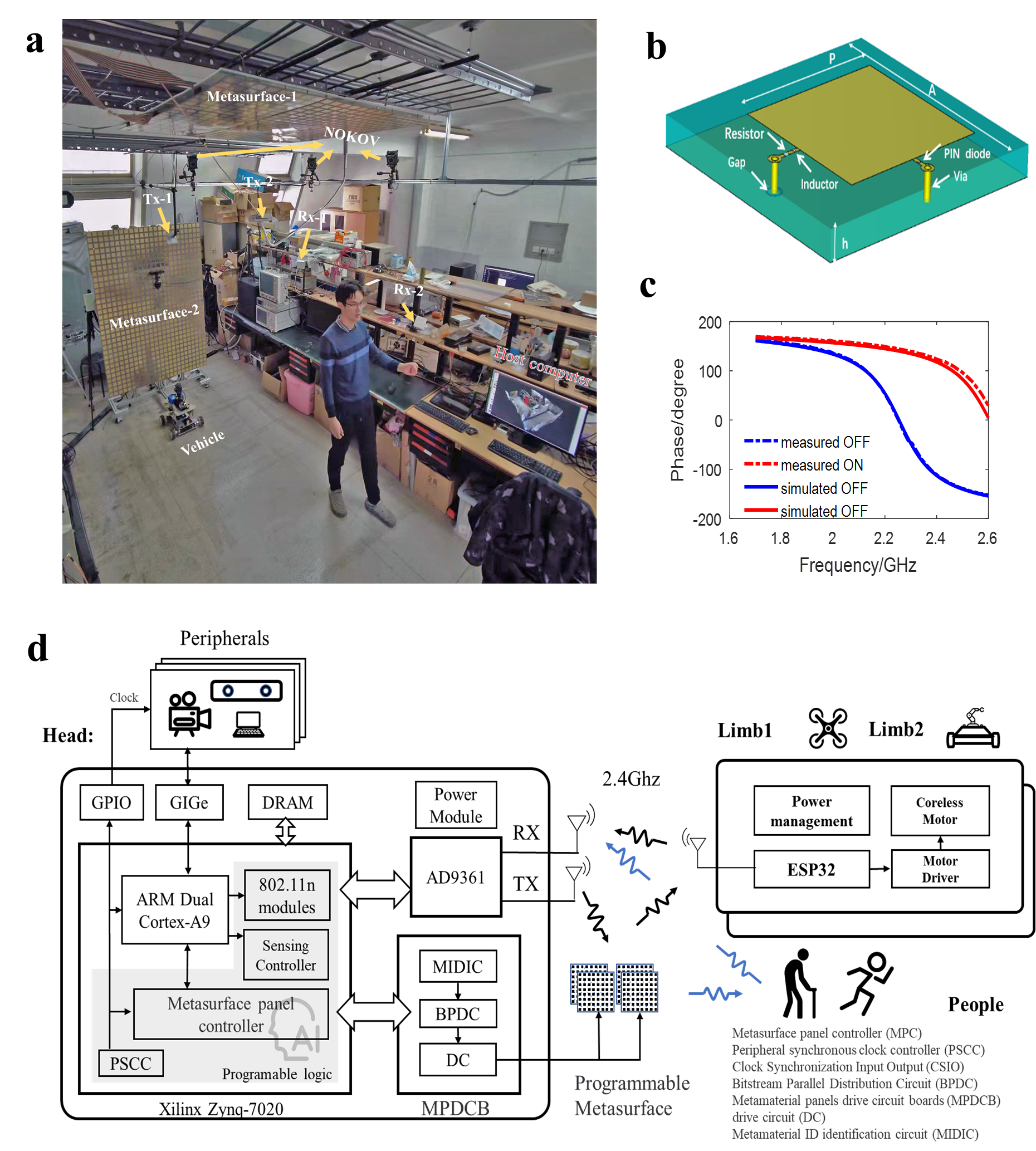


**Suppl. Fig.1**. **I2MR’s configuration. a)** Experimental setting of I2MR in room A. **b)** Schematic map of the meta-atom. **c)** Experimental and simulation results of the meta-atom’s frequency-dependent phase response. **d)** The system configuration of the I2MR, where GPIO is general-purpose IOs, and GIGe is a GIGabit Ethernet interface.

**Supplementary Note 2: More details about Fig. 1e in the main text**

On the software level, the I2MR works in a Python environment and Library functions are written in Python, in support of LabVIEW and MATLAB and other mature commercial software. The interplay between software and hardware is achieved through the MCU, Ethernet or serial communication module, etc. As demonstrated in **Fig. 1e** in the main text, all involved algorithms are managed in a four-hierarchical way: general hardware interfaces (GHI), metasurface functional modular (MFM), machine-learning-algorithms module (MLAM), and application specific module (ASFM).

The general hardware interfaces (GHI) consists of commercial peripheral driver and metasurface driver.

The GHI provides an interface for the metasurface and auxiliary peripherals (like Openwifi and NOKOV motion capture system, etc.), which is responsible for identifying the connected hardware and dealing with any abnormal operations. The commercial peripheral driver corresponds to the peripheral’s API. The device information is preprocessed and packaged for the software library of the upper hardware abstraction layer. The metasurface driver is responsible for providing the host computer control interface of the metasurface and assigning the coding sequence to the metasurface’s meta-atoms through Ethernet.

The ASFM, as implied by the name, consists of basic application-specific algorithms including basic signal processing algorithms and advanced machine learning algorithms, such as, field-focusing algorithms, object detection/localization/tracking algorithms, deep artificial networks and deep learning algorithms, path planning algorithms, and so on.

The MFM is a library of metasurface-specific algorithms for implementing the coding/decoding and bitstream transmission on the metasurface level, monitoring the metasurface’s operational status in real time, and handling any exceptions and errors, etc. When a metasurface is installed, the MFM will automatically identify it and synchronize it with the whole system.

**Supplementary Note 3: Point-cloud deep artificial neural network for converting the microwave data to a 3D point-cloud representation of the human**

Here, we present some details about the point-cloud artificial neural network for converting the measured microwave data to the desired 3D point-cloud representation of the subject. The proposed architecture of the our point-cloud network is based on a set of multilayer perceptron (MLP) networks, and has been detailed in **Suppl. Fig. 2**. The input of the network which is shown in **Suppl. Fig. 2** is divided into two main parts. One is the global prior $S=\{(x_{i}, y_{i}, z_{i})|i=1, \cdots, N\}\in\mathbb{R}^{N\times3}$, which means $N$ points are randomly selected from the lab space and $(x_{i}, y_{i}, z_{i})$ denotes the initial 3D coordinates of the $i$th point. The other input is the field matrix $E\in\mathbb{R}^{1\times d}$. It is obtained by processing the the raw microwave data $E^{sct}\in\mathbb{R}^{M\times d_{l}}$ by the Conv1D layer with Max_pooling layer, and then we replicate the matrix $E$ $N$ times. Here we set $M\times d_{l}$ as 20×128 and $d$ as 64, where 20 corresponds to the number of random coding patterns of the programmable metasurface, 128 is for sampling points of received microwave signals per pattern, and 64 is the output dimension of max pooling. Guided by the microwave information provided by $E$, the points in $S$are transformed into the point cloud of the human body $P=\{(x_{i}, y_{i}, z_{i})|i=1, \cdots, N\}\in\mathbb{R}^{N\times3}$ through coupling MLP modules.

In this work, we choose the Chamfer Distance as the loss function to find the desired 3D point cloud representation of the subject. In addition, $N$ is set as 2048 because the 3D point-cloud model of the human we collected is composed of 2048 points. We utilize fully-connected layers with the nonlinear activation function of LeakyReLU and introduce the batch normalization (BN) to further stabilize training. In addition, we use Kaiming initialization and weight normalization on the linear layers. Other training parameters are set as follows: optimization is performed using Adam method, the learning rate is 0.0001 and the batch size is set as 64. We collected more than 50,000 data sets involving diverse actions from multiple test subjects. 70% of our data set were used as training samples. The PyTorch library is used to implement our network and it takes about 300 epochs to complete the training process. A training step takes about 2 seconds on an Nvidia GTX 3090 which we used. In the test stage, the loss function value of the well-trained network is about 0.0008, indicating that the generated point cloud distribution is very close to the real value.


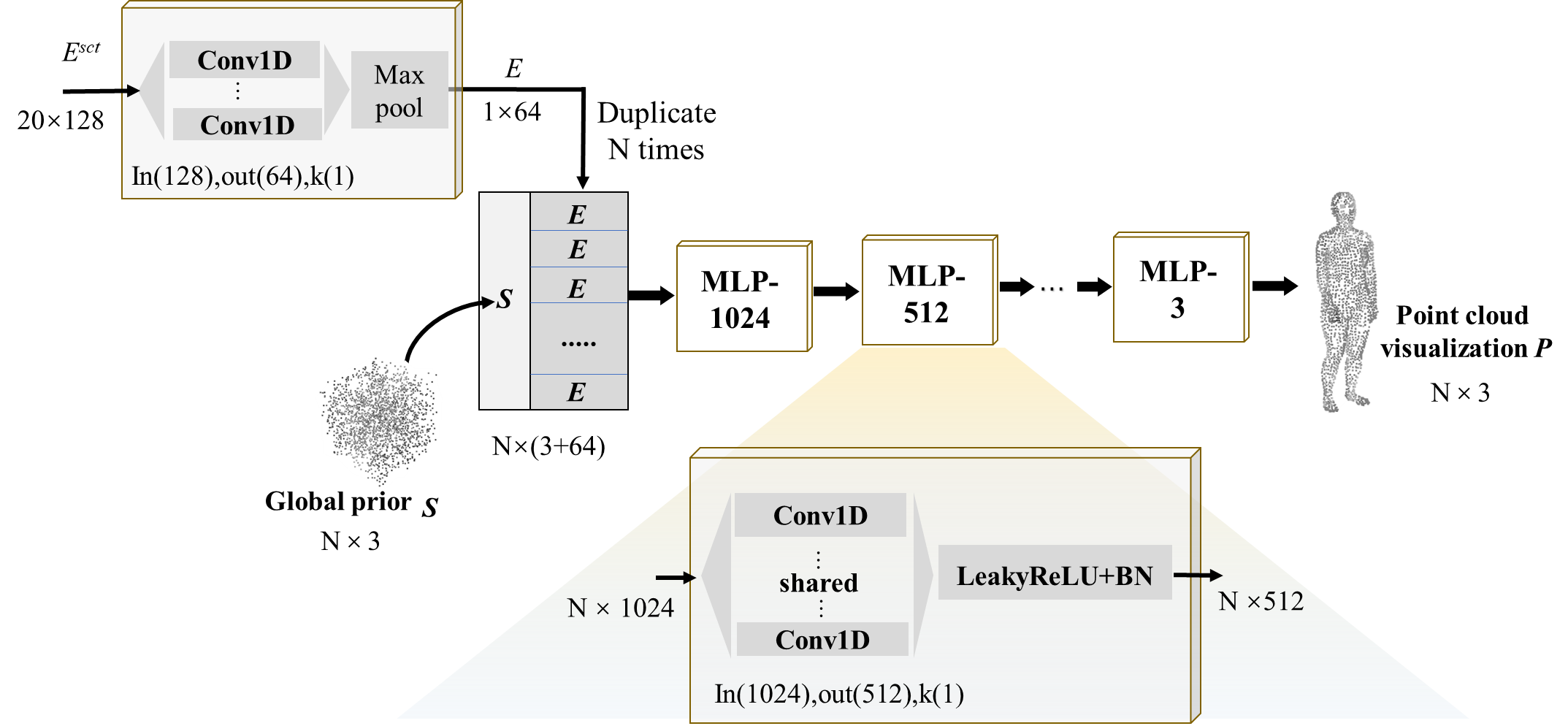


**Supplementary Figure 2 | The architecture of our point-cloud deep neural network for 3D point-cloud microwave imaging.** The input is the raw microwave data $E^{\mathrm{sct}}$ of size 20ⅹ128, where 20 is the number of coding patterns of the programmable metasurface, and 128 is the number of frequency points of the received microwave signal per pattern. The output is the desired N-point cloud representation of the subject, and each point is defined by its spatial coordinates. MLP: multi-layer perceptron network (eight MLPs are used); S: 3D-point global prior which is set to be zero-mean unit-variance Gaussian random numbers; BN: batch normalization; LeakyReLU: nonlinear activation function; Conv1D: one-dimensional (1D) convolution operator (the size of Conv1D is {In(128), out(64), k(1)}, where In(128) is the 128-length input, out(64) is the 64-length output, and k(1) is the size of 1D kernel).

**Supplementary Note 4: Localization of the robot’s limb with a complex-valued LSTM RNN**

A fundamental but important issue of the I2MR is the localization of the robot’s limb. To this end, we estimate the location of the robot’s limb by processing its ACK signals using a deep learning technique. Specifically, we designed a long short-term memory recurrent neural network (LSTM RNN), as shown in **Suppl. Fig. 3a**, which maps the complex-valued ACK signals emitted by the robot’s limb and received by the robot’s head into the location of the robot’s limb. The input has dimensions of 2×400×20, where 20 corresponds to the number of random coding patterns of the programmable metasurface, 400 represents the time-domain sampling points of the received ACK signals per control coding pattern of metasurface, and 2 stands for the real/imaginary parts per sampling point. We choose the LeakyReLU as the activation function between all FC layers and add Tanh in the LSTM Cell. The loss function is MSE (mean square error). To generate our data set, 20,000 locations were randomly selected in our indoor environment (room A, room B and corridor). 70% of our data set was used as training samples, and the rest for testing. We implement our framework using PyTorch and train it on a single NVidia GTX 3090 GPU using the Adam Optimizer with the learing rate of 0.001. In our experiment, the batch size is conventionally set to 64 and the the number of epoch is set as 500. The convergence of training and test as a function of training epochs is shown in **Suppl. Fig. 3b**, where the y-axis denotes the MSE of the estimated location. It can be seen that a localization with an average accuracy of 0.05m on is achieved.


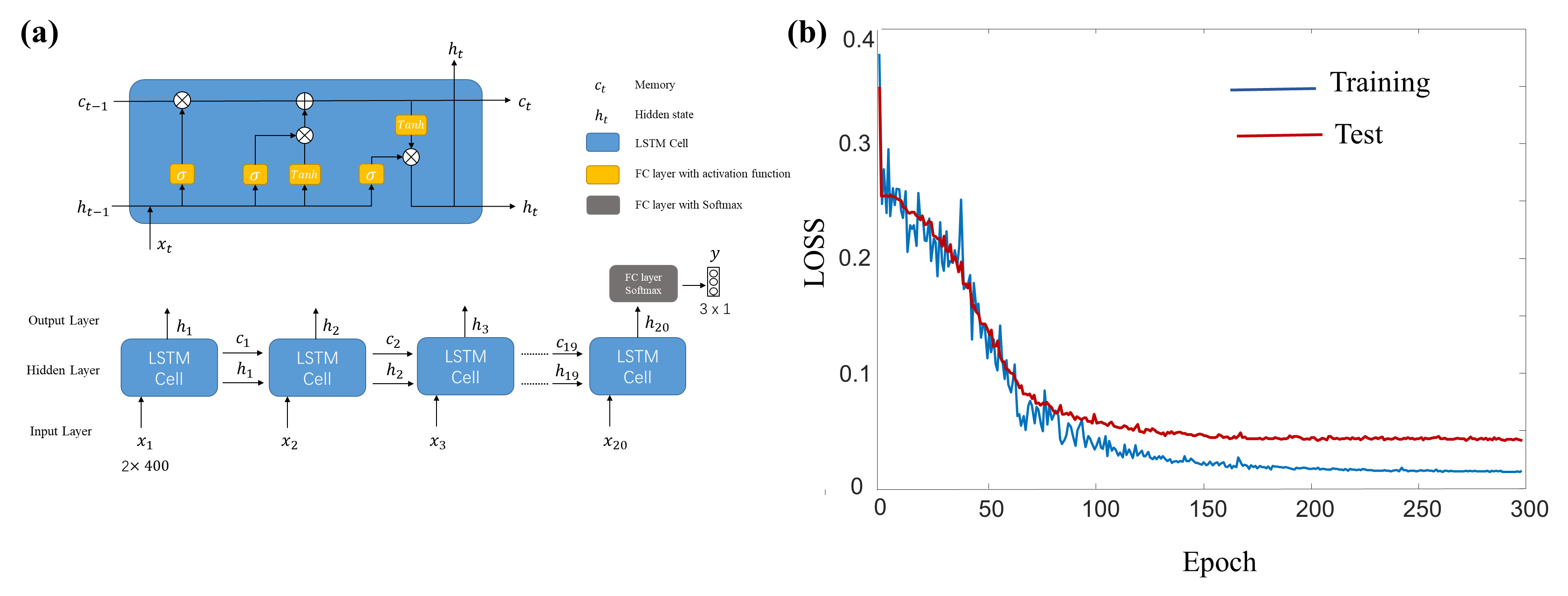


**Suppl. Fig. 3 Results of localization of robot’s limb** (**a**) The structure of the LSTM RNN for the localization of the LSTM-RNN, FC denotes fully connected layer. (**b**) The convergence behavior as the growth of the iteration epoch.

**Supplementary Note 5: Online-search algorithms for finding the optimal control coding patterns of the programmable metasurface**

As outline in main text, it is challenging to find the optimal binary coding pattern of the metasurface for focusing because the Green’s function of the complicated physical environment is not analytically available. Therefore, an online-search algorithm is developed in this work. The online-search iterative algorithm works in a trial-and-decision manner (see Table 1):

For each iteration, MⅹM adjacent meta-atoms are randomly selected and flipped; the power level of the microwave signal at the focused location is checked: if the signal’s power is increased, then the current states of the chosen meta-atoms are kept; otherwise we return to the previous configuration. We repeat this procedure until a convergence criterion is satisfied: either, no further obvious improvement of the signal power is observed, or the maximum iteration steps is reached. The initial guess of the coding pattern of the programmable metasurface is achieved by the modified G-S algorithm, where the free-space Green’s function is assumed.

Finally, we would like to remark that the optimization speed of the online searching method depends on the switching speed of metasurface and the signal measurement and feedback speed of the antenna on which we focus. The switching speed of the designed metasurface is 1us, and the measurement signal feedback speed based on the TCP protocol is 100us. Thus, the online search method will take about 0.1s per iteration.

Here, four sets of experiments are conducted to evaluate the performance of the developed online search algorithm, when the focused point is in Room A or in the Corridor. Correspondingly, the experimental signal power as a function of the iteration index is reported in **Suppl. Fig. 4a** and **b**, respectively. Recall that two programmable metasurfaces are involved, one is installed on the floor parallel to a wall (referred to as the metawall for short), and the other is installed on the ceiling (referred as metaceiling for short). We also examine in **Suppl. Fig. 4** the effect on the achievable signal power at the focusing point using only one of our two available programmable metasurfaces. Thirteen optimized coding patterns of the programmable metsurfaces, corresponding to the first 13 points of the trajectory in **Fig. 3a** in main text, are plotted in **Suppl. Fig. 4c**. The patterns are non-intuitive, evidencing the complexity of the multipath reflections in our indoor environment. It can be observed from above results that the combination of two programmable metasurfaces can give rise to the remarkable improvement on the signal power at the intended focusing location.


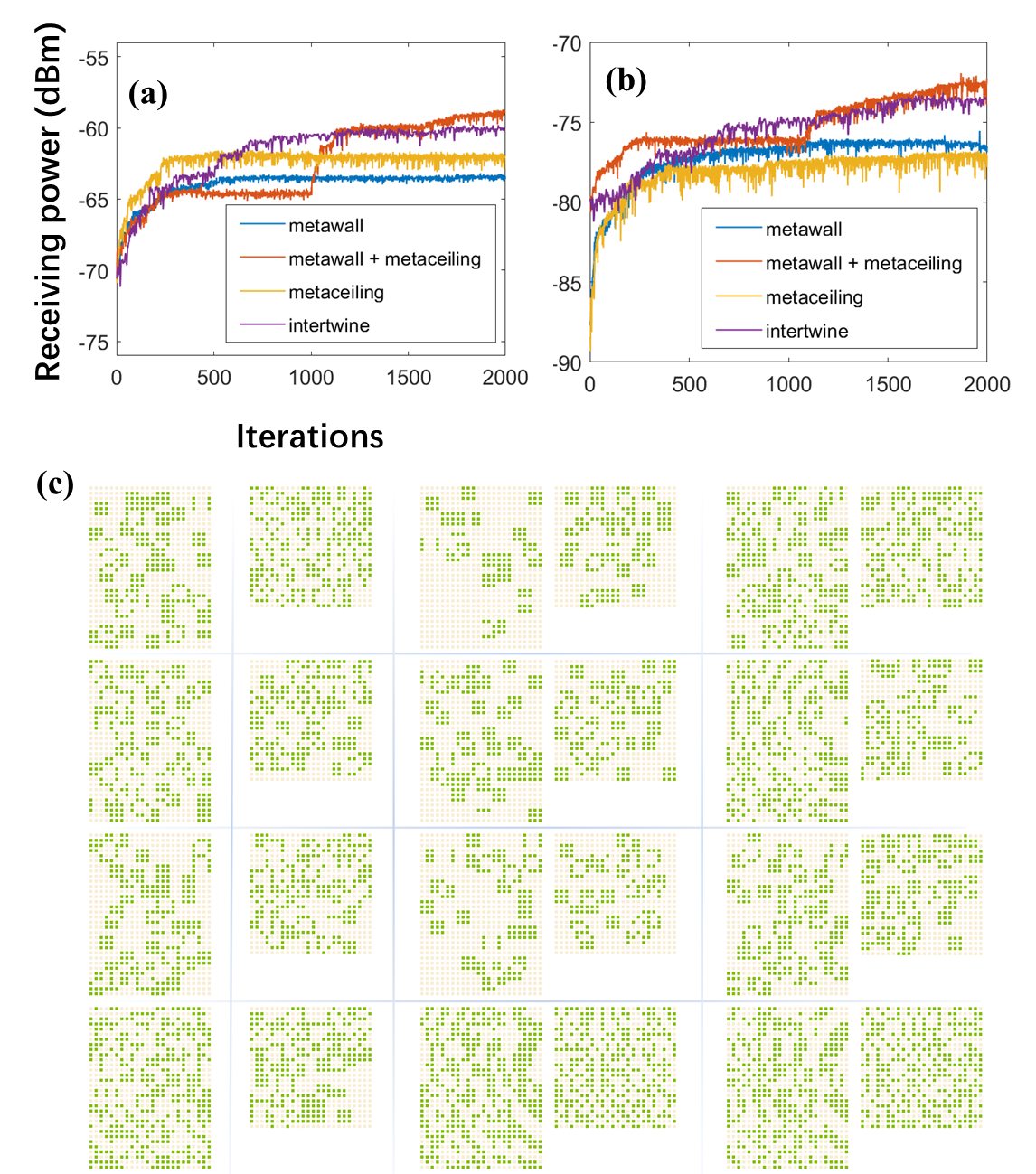


**Suppl. Fig. 4** The dependence of the focusing power on the iteration index when the intended receiver is in Room A (**a**) or in the Corridor (**b**). (**c**) The optimized coding pattern of two programmable metasurfaces, where the green squares indicate that corresponds to the meta-atom is in its ON state.

**Table1.** Online-search algorithm for finding the optimal binary coding pattern of the programmable metasurface with $N_{x}\times N_{y}$ meta-atoms. Herein, M=3, $C_{curr}$ is an 2D array with size of $N_{x}\times N_{y}$, and Get($P_{focus})$ denotes the power level of the received microwave signal at the intended location. M is a threshold.


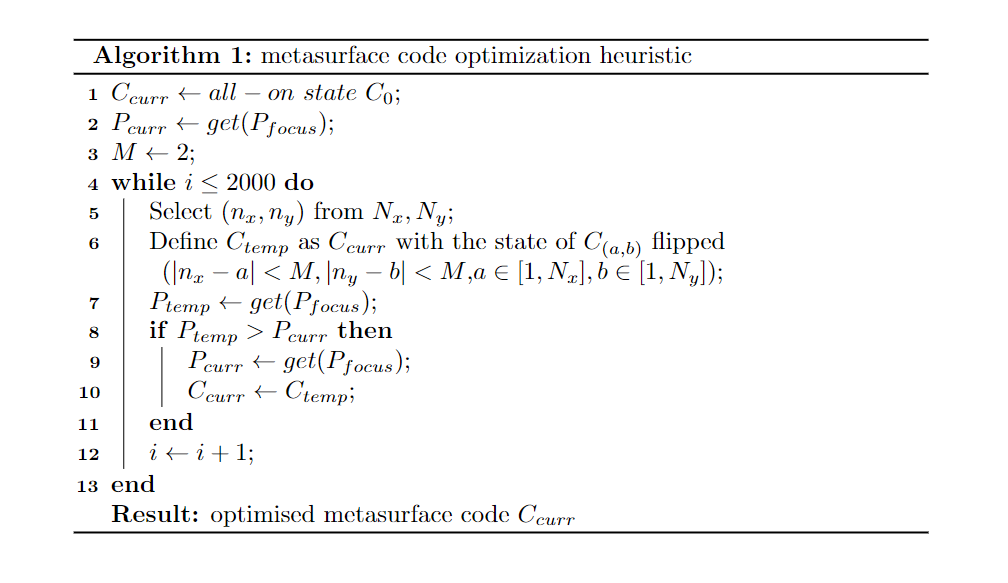


**Supplementary Note 6: Recognition of the human body gesture with I2MR**

In this section, we present additional details about the recognition of human body gestures with I2MR. As outlined in main text, I2MR is capable of ‘understanding’ the human behaviors and requests by remotely perceiving and recognizing the body gestures via in-situ microwave sensing combined with deep learning techniques. To this end, we conceive 12 body gestures for the human-robot interplay, as shown in **Suppl. Fig. 5c.** The designed body gestures are divided into two groups: one includes 9 gestures for remotely controlling the mechanical movement of robot’s limb (including start, forward, backward, left, right, height rise, stop, acceleration, and deceleration), and the other includes 3 gestures, for illustration, for recognizing the human states (stand, sit, and squat). Of course, more body gestures related to the human body behavior can be readily integrated into our I2MR.

We frame the recognition of the human body gesture as an object classification problem, and we use a deep learning technique, (a Long Short-Term Memory Recurrent Neural Network, LSTM RNN) to map the complex-valued radio signals into the label of the human body gesture. The input radio signal has dimensions of 2ⅹ100ⅹ20, where 20 corresponds to the number of random coding patterns of the metasurface, 100 represents the 100 frequency points of the received signal per coding pattern, and 2 stands for the real/imaginary parts per frequency point. The designed LSTM RNN is shown in **Suppl. Fig. 5d**. The structure of the designed LSTM RNN is divided into three parts: input layer, hidden layer and output layer; there are 64 neurons for each hidden layer. The number of the parameters in this ANN is about 9,000 in total. Other parameters are set as follows: the nonlinear activation function is Softmax, the loss function is cross-entropy Adam optimizer, the learning rate is 10^-2^, the batch size is 64, and 50 training epochs are used. To train the LSTM RNN, ten participants (3 females and 7 males) were asked to act with the 12 designated body gesture in our indoor environment, and we collected 8000 samples per gesture. We took 70% of them as training samples and the remaining 30% for testing. Experimental results for recognizing the 12 body gestures are shown in **Suppl. Fig. 5a** and **b**, when the subject is in Room A or in the Corridor, respectively. It can be observed that the classification accuracy of above 90% can be achieved by I2MR, even if the subject acts in the corridor.


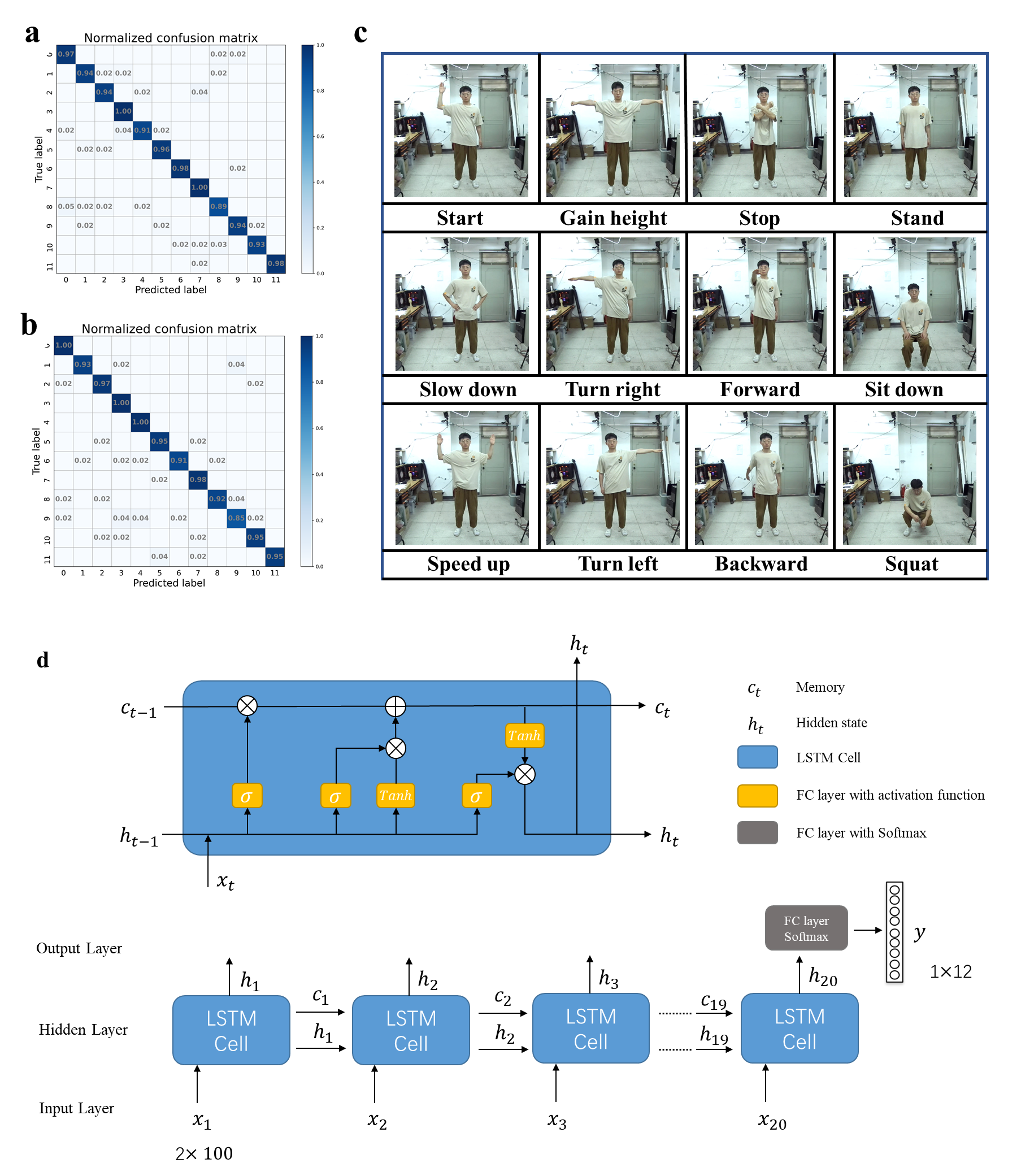


**Suppl. Fig. 5 Experimental results of human body gesture recognition.** (**a**) Classification matrix of 12 different body gesture when the subject in room A. (**b**) Classification matrix when the subject in the Corridor. (**c**) Pictures of 12 human body gestures. (**d**) Structure of the LSTM RNN. FC denotes a fully connected layer.

**Supplementary Note 7: More details about the robot’s limb**

In this supplementary note, we present additional details about the design of the robot’s limb. In principle, the robot’s limb can be realized with various edge devices free of computing and sensing capabilities, such as mobile vehicles, robotic arms, robotic dogs and smart-home devices. In our implementation, the robot’s limb is composed of a mobile vehicle free of computing and sensing capabilities with four McCollum wheels (see picture in **Suppl. Fig. 6a** and the kinematic model in **Suppl. Fig. 6c**), which has a profile of 50 mm × 30 mm × 40 mm, four McCollum wheels, a weight of 6.1 kg and a maximum driving speed of 1.2 m/s. As shown in **Suppl. Fig. 6b**, the robot’s limb has two building modules: the communication-control module and the actuator module. The communication-control module is responsible for converting the control command from the robot’s head into a set of control quantities for the actuator module. In our implementation, the communication-control module consists of an ESP32 controller. The actuator module is used to drive the actuators to perform the desired actions, and can be composed of motors, servos, displays, audio equipment, etc. Here, the actuator module is made of a MD36N 35W DC brush motor.


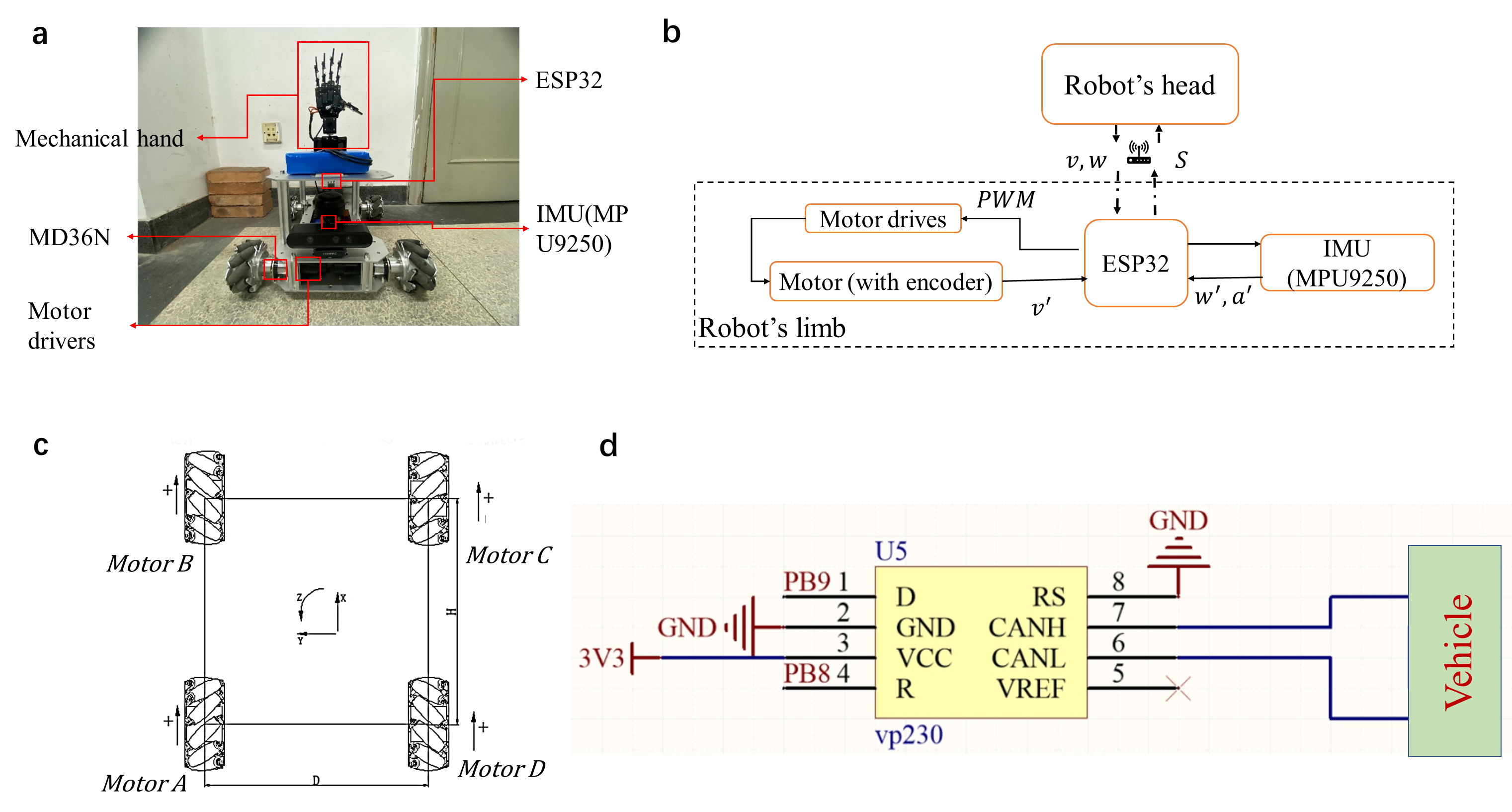


**Suppl. Fig. 6.** **System configuration of robot’s limb**. (**a**) Operational principle of the robot’s limb. (**b**) Picture of the vehicle free of sensing or computing capabilities. **c**) Kinematic model of the mobile vehicle. (**d**) Conversion between the controller ESP32 and the vehicle via the VP230 chip. (v: velocity; w, w’: angular velocity; S: limb’s state; a, a’: acceleration velocity; PWM: pulse width modulation).

Next, we detail the motion control process of the robot’s limb after receiving a motion command from the I2MR’s head. To simplify the kinematic model of the McCollum wheels, we make two assumptions: (1) the omnidirectional wheels do not slide relative to the ground while there is sufficient friction on the ground; (2) four wheels are distributed on the four corners of the rectangle and they are parallel to each other. Then, with reference to **Suppl. Fig. 6c**, the velocities of the four wheels are denoted as $V_{A}$,$V_{B}$, $V_{c}$, $V_{d}$ and can be estimated as:

$$\begin{aligned} &V_{A}=+V_{x}+V_{y}-\omega(a+b) \\ &V_{B}=+V_{x}-V_{y}-\omega(a+b) \\ &V_{C}=+V_{x}+V_{y}+\omega(a+b) \\ &V_{D}=+V_{x}-V_{y}+\omega(a+b) \end{aligned}$$

where $V_{x}$ and $V_{y}$ are the translational velocities of the vehicle along the X and Y directions, respectively, and $\omega$ is the rotational velocity of the vehicle along the Z direction. In addition, $a=\frac{D}{2}$ and $b=\frac{H}{2}$, where D and H are the wheel-base and axle-base, respectively.

Finally, we briefly discuss the procedure of the vehicle’s motion control. We first estimate the velocities of the four wheels using the above expressions and implement a PID (Proportional Integral Differential) control algorithm to control each wheel individually. Meanwhile, the speed feedback is obtained from the on-board IMU (Inertial Measurement Unit) and the encoder embedded in the motor MD36N, and the controller ESP32 communicates with the vehicle through the data conversion module VP230 chip using a so-called CAN (Controller Area Network) communication method. To this end, as shown in **Suppl. Fig. 3d** for our implementation, the pins PB8 and PB9 of the ESP32 are connected to pins D and R of the VP230, and the pins CAN/H and CAN/L of the VP230 are connected to the CANH and CANL pins on the vehicle.
